# Production of clean-label starch using physically treated starch blending and its application as a xanthan gum substitute

**DOI:** 10.1007/s10068-025-01825-y

**Published:** 2025-01-31

**Authors:** Shinjae Park, Yong-Ro Kim

**Affiliations:** 1 Department of Biosystems Engineering, Seoul National University, Seoul, 08826 Republic of Korea; 2 Center for Food and Bioconvergence, Seoul National University, Seoul, 08826 Republic of Korea; 3 Convergence Major in Global Smart Farm, Seoul National University, Seoul, 08826 Republic of Korea; 4 Research Institute of Agriculture and Life Sciences, Seoul National University, Seoul, 08826 Republic of Korea

**Keywords:** Clean label starch, Xanthan gum, Starch blending, Ultrasound, Modeling

## Abstract

This study explores clean-label starch as a functional alternative to xanthan gum in food applications. The optimal blending ratio of ultrasound-treated rice and potato starch was found to be 25:75, resulting in a product with enhanced stability and viscosity similar to xanthan gum. A mathematical model was developed to adjust the viscosity of clean-label starch to match xanthan gum at various concentrations and shear rates, showing high predictive accuracy (R^2^ = 0.988). The clean-label starch was then tested in food applications, including as a thickener in tomato ketchup. In ketchup, samples with added clean-label starch exhibited similar serum separation and viscosity reduction as those with xanthan gum. Overall, clean-label starch shows strong potential as a functional, clean-label alternative to xanthan gum, offering both economic and nutritional benefits in food products.

**Supplementary Information:**

The online version contains supplementary material available at 10.1007/s10068-025-01825-y.

## Introduction

Recently, there has been a growing global trend toward the preference for ‘‘clean label food’’ (Cao and Miao, 2023; Park and Kim, 2021). The initiative to designate foods with a clean label, signifying adherence to criteria such as the use of natural ingredients and minimal processing, originated in the United Kingdom during the 1990s (Baines and Seal, 2012). Current trends reveal an unprecedented level of consumer interest in the ingredients and manufacturing processes of food products. In other words, today’s consumers are not merely opting for 'healthy food'; rather, they are meticulously scrutinizing the ingredients of their food and evaluating the environmental impact of the products they consume (Seo et al., 2016). Consequently, the current clean label paradigm is being expanded to include the entire spectrum of food processing.

Xanthan gum (XG) is among the most widely utilized hydrocolloids, attributed to its distinctive and beneficial attributes. It is synthesized by the bacterium *Xanthomonas campestris*, which is typically found on the leaves of plants such as those belonging to the cabbage family (Afnan et al., 2023). The structural backbone of the xanthan molecule is similar to that of cellulose. However, the presence of trisaccharide side chains surrounding the primary chain gives xanthan a relatively rigid, rod-shaped structure. This unique structure imparts distinctive rheological properties, making xanthan highly useful in various industries (García-Ochoa et al., 2000). XG is valued in the food industry for its emulsion-stabilizing properties, especially in products like salad dressings and mayonnaises, where it helps create a desirable gel-like texture. Its thermal stability, compatibility with a wide range of food ingredients, and pseudoplastic rheological characteristics also significantly contribute to its extensive applications (Preichardt and Klaic, 2016). Nevertheless, XG may be associated with various side effects, including the production of intestinal gas (flatulence) and abdominal distension (bloating). People who are exposed to xanthan gum powder might experience flu-like symptoms, as well as irritation of the nasal passages and throat, and respiratory issues (Sargent et al., 1990). Additionally, hydrocolloids are categorized as ‘‘additives’’ rather than ‘‘ingredients,’’ which could make XG less suitable for the current food trend promoting clean label products.

Some alternatives to overcome these issues include the physical modification of starch, such as blending, ultrasound treatment, and pre-gelatinization. Starch is extensively utilized within the food industry for its functions as a viscosity modifier, freeze–thaw stabilizer, and emulsion stabilizer. These properties are crucial for imparting functionality to foods and improving their texture and taste (Chen et al., 2003; Jobling, 2004). These effects can be further maximized through blending, ultrasound or pre-gelatinization. Consequently, these processes enable the substitution of conventional food additives with environmentally sustainable ingredients. As previously indicated, this method aligns with contemporary consumer food trends and has the potential to produce healthier and hypoallergenic food ingredients.

This research focuses on developing clean label starch (CLS) as an alternative to XG through various physical treatment techniques such as ultrasound, blending and pre-gelatinization. Based on preliminary experimental results by Park and Kim (2023), rice and potato starches, chosen for their strong viscosity-enhancing effects when blended, were used. Regression analysis was performed to match CLS properties with those of XG, followed by a comprehensive examination of CLS’s physicochemical characteristics. CLS was then incorporated into the formulation of tomato ketchup. An investigation was conducted to assess the stability of the ketchup.

## Materials and methods

### Materials

Rice starch was extracted from native rice (*Ilmibyeo*, Korea) through an alkaline steeping method as described by Kim et al. (2010). Potato starch was acquired from a local market. Xanthan gum was generously supplied by MSC Co. (Yangsan, Korea). Concentrated tomato paste was obtained from a local retailer.

### Methods

### Production of clean label starch

Blending, ultrasound, and pre-gelatinization treatments were used to manufacture clean label starch as a replacement for xanthan gum. Binary starch blends were prepared from rice and potato starch samples at different blending ratios (100:0, 75:25, 67:33, 50:50, 33:67, 25:75, 20:80, 17:83, 0:100) and then used in the experiment. For the ultrasound treatment, 30 g of starch was dispersed in 150 mL of distilled water. During the stirring process, ultrasound waves were applied using an ultrasound probe (VC 750, Sonics, USA). The operational parameters were set to a frequency of 20 kHz and a power output of 750 W, with the treatment duration established at 30 min. Samples with the final determined mixing ratio were dispersed in distilled water at a concentration of 3 wt% and gelatinized for 20 min at 95 °C with gentle stirring. After gelatinization, the starch pastes were stored at -80 °C for 1 day to form frozen hydrogels. The hydrogels were then freeze-dried using a freeze dryer (FDS8508, Ilshin Biobase Co., Seoul, Korea) at -50 °C and 5 mTorr for 3 days. The resulting dried gel was evenly crushed using a blender and stored at room temperature.

### Pasting properties

The starch's pasting properties were evaluated with a Rapid Visco Analyser (RVA 4 Newport Sydney, Australia). Following dispersion of 3 g of starch in 25 mL of distilled water, it was kept at 50 °C for 1 min before proceeding with the experiment. The experiment involved heating to 95 °C for 7.5 min, maintaining the temperature for 2.5 min, and then gradually cooling to 50 °C for 7.5 min. During this procedure, pasting temperatures, peak viscosity, trough viscosity, and final viscosity were recorded.

### Steady-state flow test

A steady-state flow test was conducted using a rheometer (AR 1500ex, TA Instruments Ltd., USA) to simulate the viscosity of xanthan gum with clean-label starch paste. To obtain steady shear (shear stress and shear rate) data, a plate-plate geometry (20 mm diameter, 1 mm gap) was used at 25 °C, with shear rate ranging from 1 to 100 s^−1^. The steady shear rheological properties of the samples were then described by fitting the data to the well-known power law model.

1 $$ \sigma \, = \, K \, \gamma^{n} $$

where σ is the shear stress (Pa), $\dot{\upgamma }$ is the shear rate (s^−1^), K is the consistency index (Pa·s), n is the flow behavior index (dimensionless). Using the magnitudes of K and n, apparent viscosity at 50 s^−1^ was calculated.

### Morphology

Observations of the morphology of starch blends were carried out using a high-resolution optical microscope (Carl Zeiss, Axio Imager A1, Göttingen, Germany). Samples were prepared by placing a small amount of starch paste on a glass slide, followed by covering with a glass coverslip. The specimens were examined under bright-field illumination at magnifications of 400x. Care was taken to ensure consistent lighting and focus during the imaging process to allow for accurate observations.

### Regression analysis

Regression formulas were derived using SPSS (version 21.0, IBM Corp., Armonk, NY, USA) to analyze the changes after physical treatments and to correlate the viscosity of the manufactured CLS with that of XG. Non-linear regression equations were applied to predict the viscosity of XG under different shear rate conditions.

### Statistical analysis

All data are expressed as the mean ± standard deviation. Statistical analyses were conducted using SPSS for Windows (version 21.0, IBM Corp., Armonk, NY, USA). A one-way analysis of variance (ANOVA) was performed, followed by Duncan's multiple range test, to determine statistical significance with a threshold of p < 0.05.

## Results and discussion

### Production of clean label starch

#### Ultrasound treatment

Ultrasound treatment is a primary physical method employed for altering the characteristics of starch (Zhu, 2015). The degree of alteration in starch is influenced by the duration, temperature, frequency, and intensity of ultrasound treatment, as well as the starch's properties. Table 1 presents the pasting property data for rice and potato starch, highlighting the differences before and after ultrasound treatment. From these results, it was confirmed that peak viscosity (PV) increased after ultrasound treatment for both rice starch (from 4655 ± 209.0 cP to 5275 ± 229.2 cP) and potato starch (from 11,685 ± 586.2 cP to 17,176 ± 871.1 cP), demonstrating that ultrasound treatment could significantly enhance the thickening ability of these starches. Notably, this increase in viscosity was sustained through to the final viscosity (FV), indicating that the ultrasound-treated starches retained their structural integrity over the heating and cooling phases(Carmona‐Carmona-García et al., 2016; Chan et al., 2010). It is also noteworthy that both the viscosity and the stability ratio of the starches improved after ultrasound treatment. The stability ratio of paste refers to the ratio of viscosity at the onset of cooling to the maximum viscosity achieved before cooling (Tsakama et al., 2010). The stability ratio rose from 0.396 to 0.436 for rice starch and from 0.119 to 0.139 for potato starch after ultrasound treatment. This improvement in the stability ratio suggests that the starch paste may become more resistant to shearing forces, enhancing its ability to withstand mechanical stress during processing. Furthermore, the observed increase in FV for ultrasound-treated starch indicates that, after the initial swelling and collapse of granules, the solubilized starch components were more capable of re-associating and forming a cohesive network during cooling (Sit et al., 2014). This effect may also be due to increased amylose leaching, which is a result of ultrasound-induced alterations in the internal structure of the starch granules. Rice starch (5.2 ± 0.9% amylose) and potato starch (25.2 ± 5.2% amylose) were used in this study, and the greater amylose availability not only enhances the viscosity but may also improve the gel strength and cohesiveness of the starch, making it highly beneficial in applications that require stable and resilient gels (Biduski et al., 2018). These ultrasound-induced modifications underline the potential of ultrasound as a useful technique to modify starch functionality, improving its performance as a thickening and gelling agent in various food systems.

**Table 1:** Pasting properties of starch before and after ultrasound treatment. Significant differences in the values after ultrasound treatment for each starch are indicated as ^*^ *p* < 0.05 and ^**^ *p* < 0.01

Type		Paste temperature [ °C ]	Peak viscosity [cP]	Trough viscosity [cP]	Final viscosity [cP]	Stability ratio [-]
Rice	Native	67.3 ± 1.3	4655 ± 209.0	1843 ± 195.5	2813 ± 122.7	0.396
	Ultrasound	65.4 ± 2.1	5275 ± 229.2^*^	2299 ± 175.4^*^	3138 ± 172.3	0.436
Potato	Native	66.3 ± 1.1	11,685 ± 586.2	1386 ± 203.1	4809 ± 301.5	0.119
	Ultrasound	59.7 ± 1.9^**^	17,176 ± 871.1^**^	2382 ± 271.1^**^	5359 ± 288.7	0.139

#### Blending ratio determination and the production of CLS

From the previous study by Park and Kim (2023), it was observed that blending rice and potato starches could result in a sharp increase in viscosity. To understand this phenomenon, the blending characteristics of rice and potato starch were examined at different ratios. Additionally, all samples were pre-treated with ultrasound, as it reduces starch crystallization and increases swelling power (Carmona‐García et al., 2016), leading to a further increase in viscosity.

**Fig. 1:**
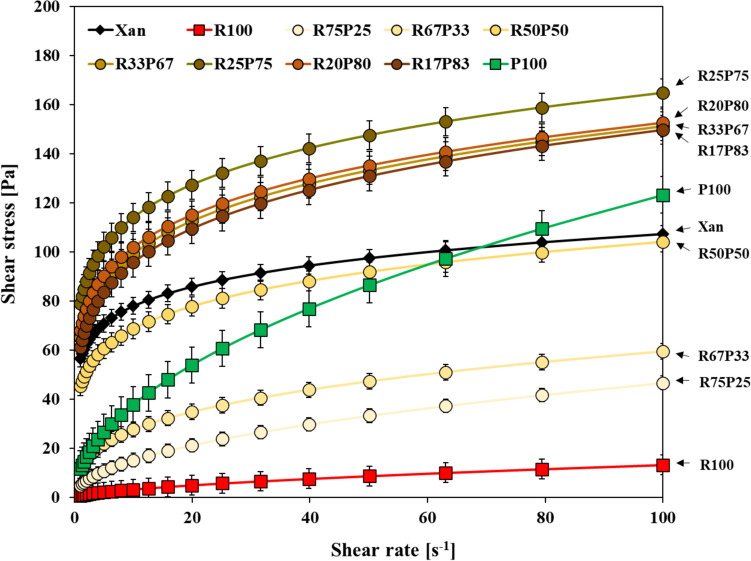
Steady-state flow curve by blending ratio of ultrasound treated rice and potato starch

Figure 1 presents the results of the steady flow tests conducted on ultrasound-treated rice-potato starch blends at a concentration of 3 wt%. Notably, the combination of rice and potato starches in equal amounts (R50P50) exhibited a significant synergistic effect, particularly at low shear rates (below 50 s⁻^1^). This synergy was evidenced by the fact that the shear stress of the starch blend was higher than that of either rice or potato starch alone, highlighting a marked improvement in the blend’s resistance to flow under applied force. Additionally, the yield stress showed a substantial increase, suggesting enhanced structural framework within the blend (Coussot, 2014). Interestingly, when the proportion of potato starch in the blend was increased relative to rice starch, the synergistic effect on viscosity became even more pronounced. The data reveal that the viscosity-enhancing effect was optimized at a rice-to-potato starch ratio of 25:75. However, beyond this ratio, as the potato starch content continued to increase, the synergistic effect began to diminish. This optimal ratio of 25:75 for maximizing viscosity is likely due to the “packing density” effect, which refers to the arrangement and compactness of particles within a material. When granular particles are combined, packing density has a significant influence on properties like flowability, suspension rheology, porosity, permeability, and mechanical strength (Jung et al., 2009; Kwan and Chen, 2012; Li and Park, 1998). Packing density can be inferred by the void content within the mixture.

**Fig. 2:**
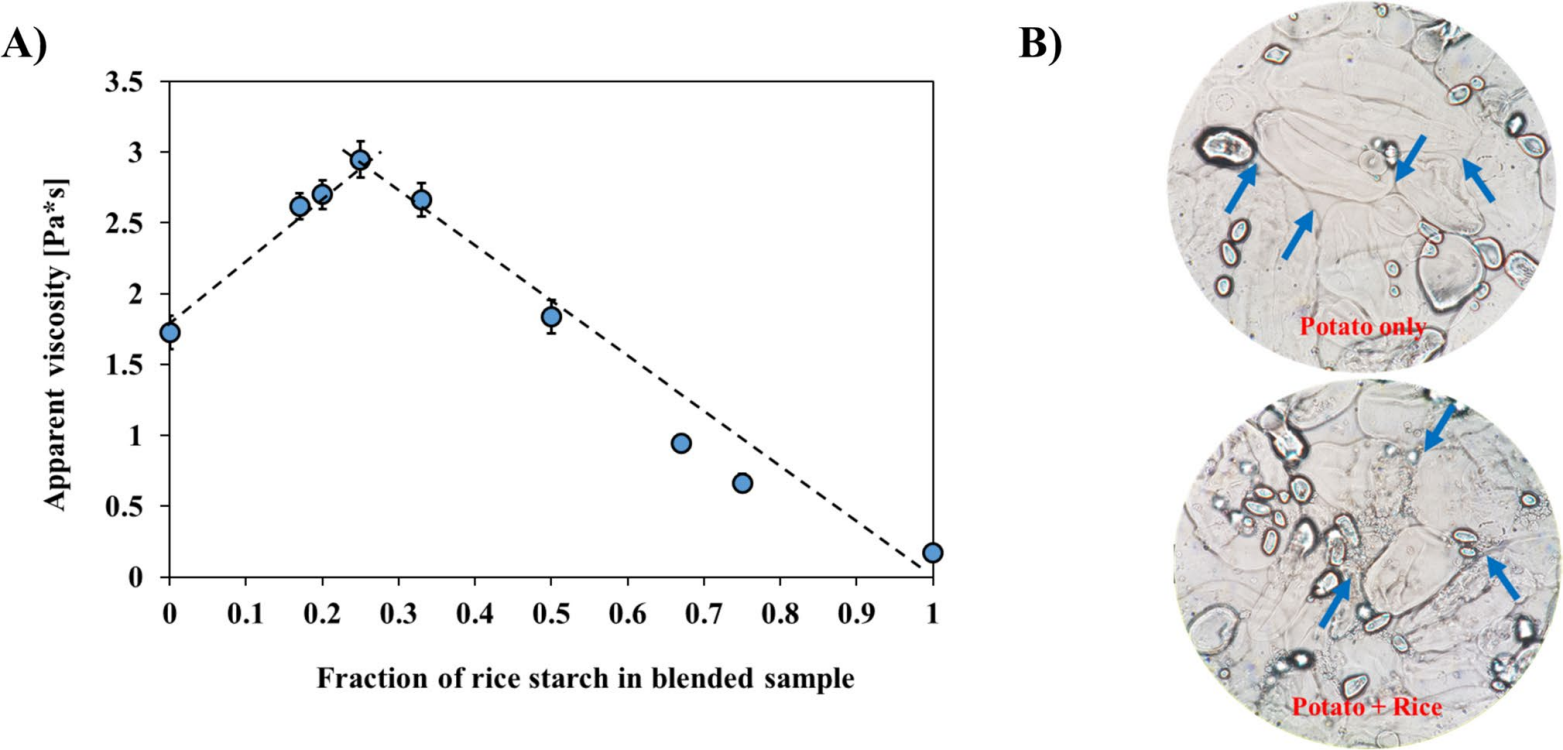
Apparent viscosity (at 50 s.^−1^) of rice-potato starch blends by blending ratio (**A**) and microscopic images of them (**B**)

In fact, Fig. 2 shows the highest viscosity was indeed achieved at the 25:75 rice-to-potato starch ratio. This finding suggested that the rice starch granules filled the voids between the larger potato starch granules, thereby reducing the empty spaces seen when only potato starch is present as observed by a light microscope (Fig. 2B). The rice starch in these voids likely generates frictional forces during shearing, which increases the mixture's overall viscosity (Hossen et al., 2011). Wong and Kwan (2014) demonstrated that when two types of particles with different sizes are blended, the highest packing density is achieved with a small-to-large blending ratio of approximately 30:70, particularly when the particle size ratio is around 0.28. The results observed in this study appear to follow a similar trend, as the maximum viscosity (i.e., packing density) was reached at a rice-to-potato starch ratio of 25:75. This is consistent with the particle size ratio of rice starch (5.17 µm) to potato starch (25.21 µm), which is approximately 0.2, thus supporting the concept that an ideal mixing ratio of small to large particles can enhance the packing density and viscosity of starch blends.

To produce CLS as a replacement for XG, ultrasound-treated rice and potato starches were blended at the optimum ratio of 25:75, gelatinized, and freeze-dried. This process enabled the CLS to dissolve easily in cold water like XG, forming a viscous paste with similar consistency to XG without extra heat treatment.

#### Viscosity adjustment

In general, starch viscosity increased exponentially with concentration, whereas XG viscosity increased in a more linear manner. Therefore, to make CLS a viable industrial alternative to XG, it is necessary to adjust its viscosity to match that of XG at varying concentrations. Referring to previous study (Park and Kim, 2023), the curve estimation technique was applied to determine the relationship between the apparent viscosity of CLS and XG at a shear rate of 50 s⁻^1^, resulting in the following equation:

2 $$ {\text{Y}} = \, 0.573 \, \times \, \ln \, \left( {\text{X}} \right) \, + \, 1.627 $$

where X is the XG concentration and Y is the CLS concentration. However, the apparent viscosity in steady-state flow tests depends on the shear rate, as both CLS and XG are non-Newtonian materials. Therefore, the above formula needs to include terms for the shear rate. Consequently, a relational formula that incorporates the viscosities of both CLS and XG, as well as the shear rate, was derived as follows:

3 $$  \begin{aligned}   Y &  = {\mkern 1mu} \left( { - 0.023{\mkern 1mu}  \times {\mkern 1mu} \ln {\mkern 1mu} \left( {SR} \right){\mkern 1mu}  + {\mkern 1mu} 0.662} \right){\mkern 1mu}  \\     \times {\mkern 1mu} \ln {\mkern 1mu} \left( X \right){\mkern 1mu}  - {\mkern 1mu} 0.055{\mkern 1mu}  \times {\mkern 1mu} \ln {\mkern 1mu} \left( {SR} \right){\mkern 1mu}  + {\mkern 1mu} 1.837 \\  \end{aligned}   $$

where X is the XG concentration, Y is the CLS concentration, and SR is the shear rate. This equation provides the concentration of CLS required to achieve a desired viscosity level equivalent to that of XG at a given shear rate. For example, to determine the CLS concentration that matches the viscosity of a 2% XG solution at a shear rate of 30 s⁻^1^, substitute 30 for SR and 2 for X, yielding a Y value of 2.06. This means that the viscosity of a 2.06 wt% CLS solution is comparable to that of a 2 wt% XG solution at a shear rate of 30 s⁻^1^.

**Fig. 3:**
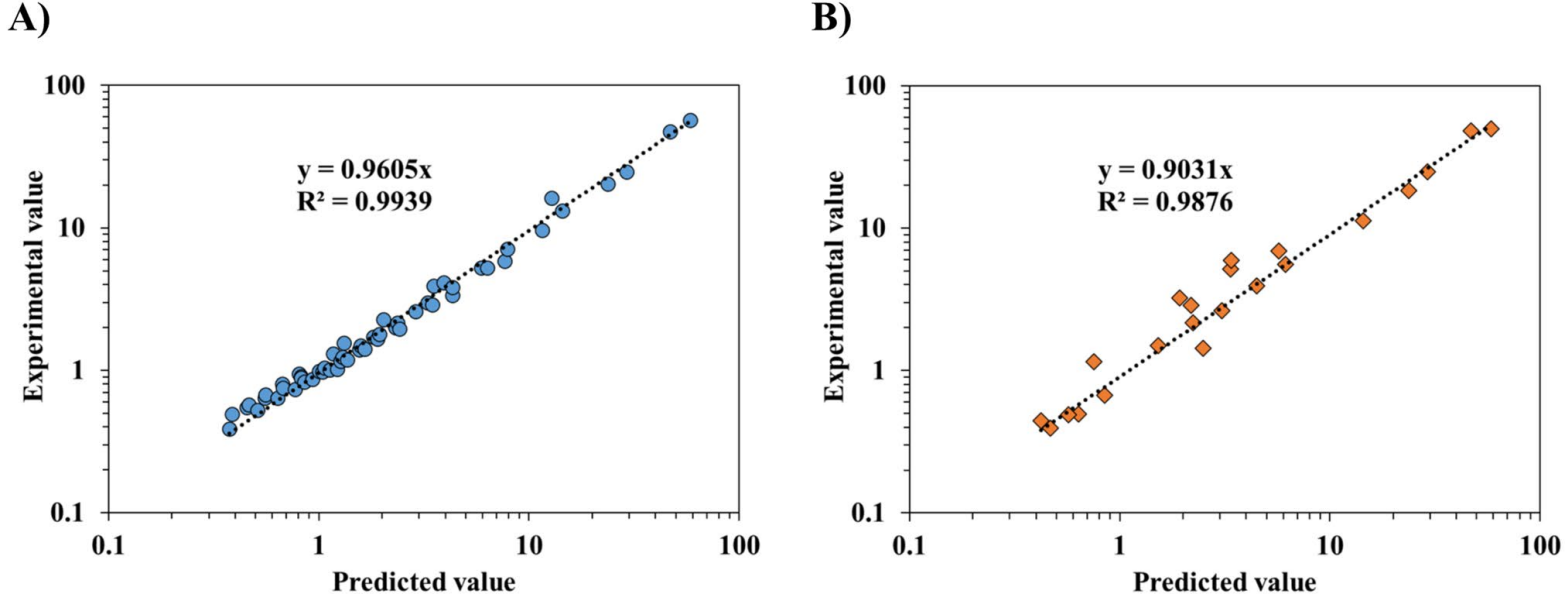
Predicted-experimental plotting for matching the viscosity of clean label starch and xanthan gum. (**A**: modeling part, **B**: validation part)

To evaluate the predictive capability of the developed equation (Eq. 3), the predicted values were compared with actual experimental values. Figure 3 shows a predicted-experimental scatter plots of modeling and validation parts. In the modeling part, experimental data on the viscosities of CLS and XG at various shear rates were used to construct the regression equation. The strong alignment between observed data and predictions indicated the model’s accuracy in capturing the viscosity relationship as a function of shear rate and concentration. Validation part then provides the predicted CLS concentrations needed to match the viscosity of XG at specific shear rates not used in the modeling stage, thereby demonstrating the accuracy of the equation developed. Since the slope of the trendline between the predicted and experimental values in validation part was approximately 0.903, and the coefficient of determination (R^2^) was calculated to be 0.988 (Fig. 3B), it was concluded that CLS could effectively mimic the viscosity of XG under different conditions.

The strong correlation between predicted and experimental data demonstrates the effectiveness of the developed equation in replicating the non-Newtonian behavior of XG. However, limitations in the model include a relatively small dataset and variability in starch properties, which can differ based on regional, seasonal, and processing factors. To improve the model’s accuracy, future research should incorporate a larger, more diverse dataset that accounts for these variations. Additionally, the influence of external factors such as temperature and storage conditions on viscosity should be studied. Nevertheless, these findings are significant as they demonstrate the potential of CLS to replicate XG's characteristics through the synergistic effects of physical processing, achieving both 'economic' and 'clean label' status.

Applications

Thus far, the manufacturing process of CLS has been explored as a potential substitute for XG, a widely used thickening agent in food products. To evaluate CLS's ability to replicate the industrial characteristics of XG, it was tested in XG-based food products, with tomato ketchup being selected as a representative example due to the common use of XG to achieve its complex texture and stability (Omidbakhsh amiri E et al.., 2015). This application provided insight into CLS’s potential to match the performance of XG in texture and quality, supporting its use in clean-label formulations.

The physical properties of ketchup have a significant impact on user convenience, making it crucial to control them effectively. Ketchup is generally known to contain around 0.5% xanthan gum (Koocheki et al., 2009). In this experiment, simple tomato ketchup was prepared by mixing concentrated tomato paste with either XG solution or CLS solution, and the stability of each mixture was evaluated. Distilled water without a thickener was added as a control.

**Fig. 4:**
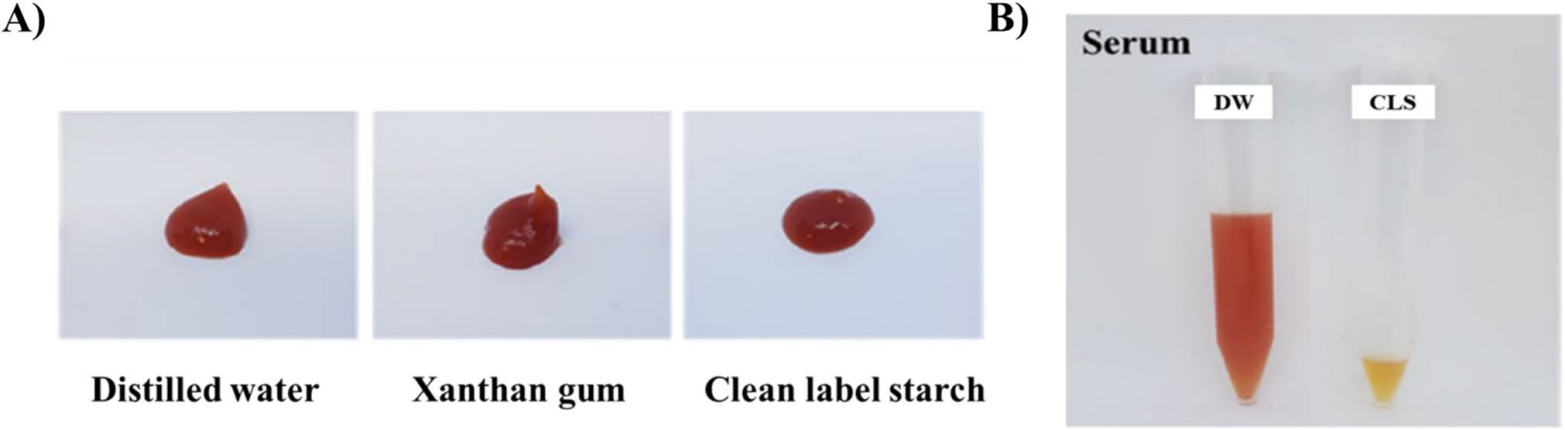
Application of clean label starch in ketchup. (**A**: appearance, **B**: stability from serum separation)

Figure 4 illustrates the appearance and extent of serum separation in the manufactured tomato ketchup samples. Initially, there was no visible difference in appearance between the control group and the samples with added XG and CLS immediately after production. However, a slight thickening of the surface texture was observed in the XG and CLS samples, suggesting that these additives may enhance the initial stability and consistency of the ketchup formulation. Following centrifugation (315xg, 10 min), significant differences in serum separation were observed (Fig. 4B). In the control group, a notable 36.8% of the initial sample weight separated as serum, indicating a lack of internal cohesion that allowed considerable water loss (Song et al., 2024). In contrast, the XG and CLS samples exhibited minimal serum separation, with only 0.0% and 1.9% of the initial sample weight separating, respectively. These results suggest that the superior stabilizing properties of both XG and CLS, which effectively maintain the ketchup matrix's integrity. This stabilization reduces serum separation, ensuring improved product consistency, enhanced structural integrity, and a more desirable texture.

**Fig. 5:**
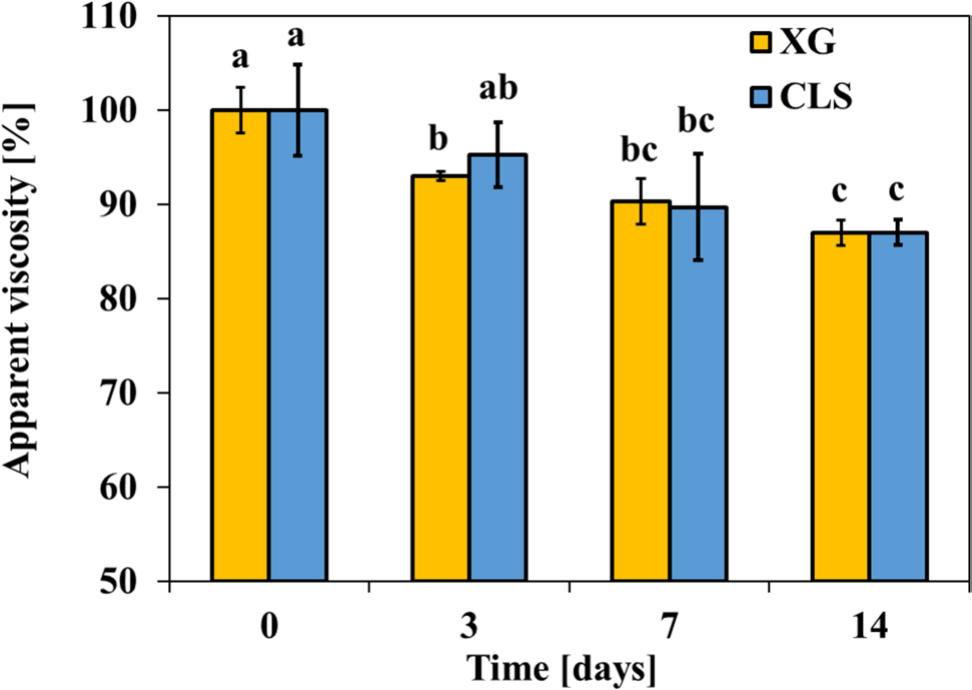
The apparent viscosities that change during the 2 weeks storage at 40 °C (XG = xanthan gum, CLS = clean label starch). Letters in the figure indicate significant differences (*p* < 0.05)

Moreover, after two weeks of storage at 40˚C, the viscosity reduction observed in the CLS-added sample was nearly identical to that in the XG-added sample (Fig. 5). This outcome indicates that CLS not only matches XG in preventing serum separation but also demonstrates comparable efficacy in preserving the rheological properties of the ketchup over time. Such performance underlines CLS's ability to maintain product stability during extended storage, a critical factor in ensuring quality in food formulations. The ability of CLS to provide these functional benefits positions it as a viable and effective stabilizer in food formulations where structural stability, consistency, and consumer-pleasing attributes are essential. Consequently, CLS offers a promising clean-label alternative to traditional stabilizers like XG, aligning with industry trends and consumer preferences for transparent and natural ingredient labeling.

In conclusion, this study demonstrated the potential of CLS as a functional alternative to XG in food products. Ultrasound treatment effectively enhanced the properties of rice and potato starch, leading to increased peak and final viscosities as well as improved stability ratios. By blending ultrasound-treated rice and potato starch at an optimal 25:75 ratio, CLS with excellent viscosity and gel-forming capabilities comparable to XG was produced. The manufactured CLS was easily dissolved in cold water, forming a viscous paste comparable to freshly gelatinized starch without additional heat treatment. To match the viscosity of XG at different concentrations, a predictive equation was derived, demonstrating the ability of CLS to replicate XG's viscosity under varying shear rates. The CLS's performance was validated against experimental data, showing a high degree of accuracy. In application, CLS was successfully incorporated into food products like tchoomato ketchup, where it showed promising results. In ketchup, CLS demonstrated comparable stability to XG, with minimal serum separation and viscosity reduction, confirming its suitability as a thickening agent.

Overall, CLS offers an effective, economically viable, and clean-label alternative to XG, with applications extending to various food products. These findings highlight the potential for CLS to become a widely used ingredient in the food industry, offering both functional and health benefits.

## Supplementary Information

Below is the link to the electronic supplementary material.

Supplementary file1 (DOCX 543 KB)

## References

[CR1] Afnan U, Sharma K, Sehgal R, Kumar V. Xanthan gum-based nanocarriers for therapeutic delivery. pp. 333-365. In: Polymeric Nanosystems. Elsevier (2023)

[CR2] Baines D, Seal R. Natural food additives, ingredients and flavourings. Elsevier (2012)

[CR3] Biduski B, da Silva WMF, Colussi R, El Halal SLdM, Lim L-T, Dias ÁRG, da Rosa Zavareze E. Starch hydrogels: The influence of the amylose content and gelatinization method. International journal of biological macromolecules 113: 443-449 (2018) 29486261 10.1016/j.ijbiomac.2018.02.144

[CR4] Cao Y, Miao L. Consumer perception of clean food labels. British Food Journal 125: 433-448 (2023)

[CR5] Carmona‐García R, Bello‐Pérez L, Aguirre‐Cruz A, Aparicio‐Saguilán A, Hernández‐Torres J, Alvarez‐Ramirez J. Effect of ultrasonic treatment on the morphological, physicochemical, functional, and rheological properties of starches with different granule size. Starch‐Stärke 68: 972-979 (2016)

[CR6] Chan H-T, Bhat R, Karim AA. Effects of sodium dodecyl sulphate and sonication treatment on physicochemical properties of starch. Food Chemistry 120: 703-709 (2010)

[CR7] Coussot P. Yield stress fluid flows: A review of experimental data. Journal of Non-Newtonian Fluid Mechanics 211: 31-49 (2014)

[CR8] García-Ochoa F, Castro EG, Santos V. Oxygen transfer and uptake rates during xanthan gum production. Enzyme and microbial technology 27: 680–690 (2000) 10.1016/s0141-0229(00)00272-6 11064050

[CR9] Hossen MS, Sotome I, Takenaka M, Isobe S, Nakajima M, Okadome H. Effect of particle size of different crop starches and their flours on pasting properties. Japan Journal of Food Engineering 12: 29-35 (2011)

[CR10] Chen Jj, Lai VMF, Lii Cy. Effects of compositional and granular properties on the pasting viscosity of rice starch blends. Starch‐Stärke 55: 203-212 (2003)

[CR11] Jobling S. Improving starch for food and industrial applications. Current Opinion in Plant Biology 7: 210-218 (2004) 15003223 10.1016/j.pbi.2003.12.001

[CR12] Jung S, Ehlert S, Mora J-A, Kraiczek K, Dittmann M, Rozing GP, Tallarek U. Packing density, permeability, and separation efficiency of packed microchips at different particle-aspect ratios. Journal of Chromatography A 1216: 264-273 (2009) 19091319 10.1016/j.chroma.2008.11.073

[CR13] Kim JM, Song JY, Shin M. Physicochemical properties of high amylose rice starches purified from Korean cultivars. Starch‐Stärke 62: 262-268 (2010)

[CR14] Koocheki A, Ghandi A, Razavi SM, Mortazavi SA, Vasiljevic T. The rheological properties of ketchup as a function of different hydrocolloids and temperature. International Journal of Food Science & Technology 44: 596-602 (2009)

[CR15] Kwan AK, Chen JJ. Roles of packing density and water film thickness in rheology and strength of cement paste. Journal of Advanced Concrete Technology 10: 332-344 (2012)

[CR16] Li Y, Park C-W. Permeability of packed beds filled with polydisperse spherical particles. Industrial & engineering chemistry research 37: 2005-2011 (1998)

[CR17] Omidbakhsh amiri E, Nayebzadeh K, Mohammadifar MA. Comparative studies of xanthan, guar and tragacanth gums on stability and rheological properties of fresh and stored ketchup. Journal of Food Science and Technology 52: 7123-7132 (2015)

[CR18] Park S, Kim Y-R. Clean label starch: production, physicochemical characteristics, and industrial applications. Food Science and Biotechnology 30: 1-17 (2021) 33552613 10.1007/s10068-020-00834-3 PMC7847421

[CR19] Park S, Kim Y-R. Development of non-linear prediction model for starch blending. LWT 190: 115567 (2023)

[CR20] Preichardt LD, Klaic PMA. Xanthan gum application in food. BUTLER, M (2016)

[CR21] Sargent EV, Adolph J, Clemmons MK, Kirk GD, Pena BM, Fedoruk MJ. Evaluation of flu-like symptoms in workers handling xanthan gum powder. J Occup Med 32: 625-30 (1990) 2391577 10.1097/00043764-199007000-00014

[CR22] Seo S, Ahn H-K, Jeong J, Moon J. Consumers’ attitude toward sustainable food products: Ingredients vs. Packaging. Sustainability 8: 1073 (2016)

[CR23] Sit N, Misra S, Deka SC. Yield and functional properties of taro starch as affected by ultrasound. Food and Bioprocess Technology 7: 1950-1958 (2014)

[CR24] Song R, Wang X, Johnson M, Milne C, Lesniak‐Podsiadlo A, Li Y, Lyu J, Li Z, Zhao C, Yang L. Enhanced strength for double network hydrogel adhesive through cohesion‐adhesion balance. Advanced Functional Materials: 2313322 (2024)

[CR25] Tsakama M, Mwangwela A, Manani T, Mahungu N. Physicochemical and pasting properties of starch extracted from eleven sweet potato varieties. African Journal of Food Science and Technology 1: 090-098 (2010)

[CR26] Wong V, Kwan A. A 3-parameter model for packing density prediction of ternary mixes of spherical particles. Powder technology 268: 357-367 (2014)

[CR27] Zhu F. Impact of ultrasound on structure, physicochemical properties, modifications, and applications of starch. Trends in Food Science & Technology 43: 1-17 (2015)

