# Supplementary material for: Production of clean-label starch using physically treated starch blending and its application as a xanthan gum substitute
Source: Food Sci Biotechnol. 2025 Jan 31;34(9):1949–56. doi: 10.1007/s10068-025-01825-y (PMC11972251; doi:10.1007/s10068-025-01825-y)
Supplement: Supplementary file 1 — Supplementary file1 (DOCX 543 KB) [file 10068_2025_1825_MOESM1_ESM.docx]

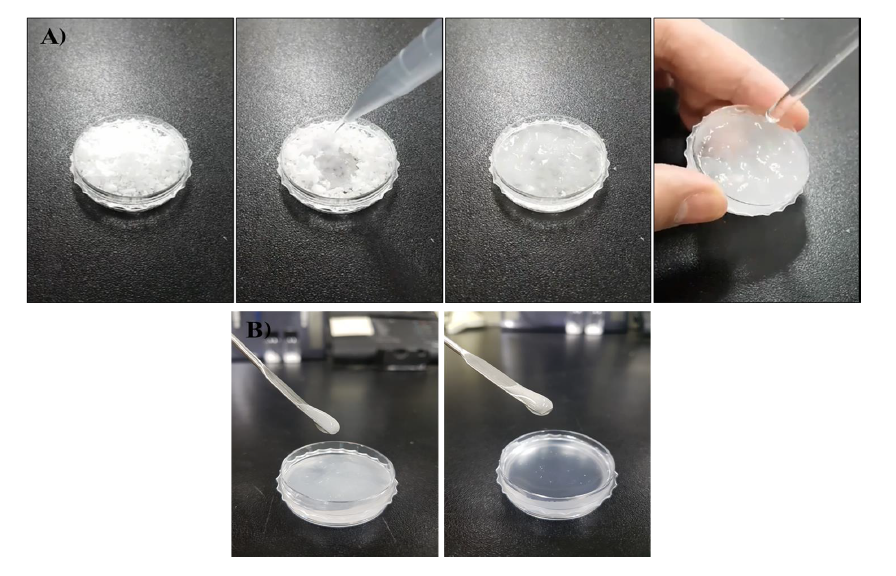
 **Rehydration of manufactured clean label starch (A) and comparison with xanthan gum (B; left: clean label starch, right: xanthan gum).**
